# A case report of curative distal gastrectomy for stage IV gastric cancer after chemoradiotherapy in a patient with a gastrojejunal gastric bypass

**DOI:** 10.1186/s40792-016-0259-x

**Published:** 2016-11-11

**Authors:** Masataka Shimonosono, Sumiya Ishigami, Takaaki Arigami, Yoshikazu Uenosono, Yasuto Uchikado, Yoshiaki Kita, Yuko Kijima, Hiroshi Kurahara, Yuko Mataki, Kosei Maemura, Shoji Natsugoe

**Affiliations:** Department of Surgical Oncology and Digestive Surgery, Graduate School of Medicine, Kagoshima University, Sakuragaoka 8-35-1, Kagoshima, 890-8520 Japan

**Keywords:** Stomach neoplasms, Gastroenterostomy, Gastrectomy, Chemoradiotherapy

## Abstract

**Background:**

Advanced gastric cancer in the lower third of the stomach often results in stricture of the gastric cavity and digestive symptoms. Gastrojejunostomy has been suggested to improve such symptoms, and the advent of new anticancer agents for gastric cancer has improved the response rate of the disease, which makes it possible to perform R0 gastrectomy in part of patients with stage IV gastric cancer. We experienced a rare case in which a patient with stage IV gastric cancer and cancerous pyloric stenosis was treated with R0 surgery after undergoing a gastrojejunal bypass procedure and multidisciplinary treatment. There have not been any previous reports about cases in which a previous gastrojejunostomy was utilized as a reconstruction route during distal gastrectomy in a patient with gastric cancer that had been treated with chemotherapy and/or CRT.

**Case presentation:**

An 80-year-old female with advanced gastric cancer and pyloric stenosis was admitted to Kagoshima University Hospital. As peritoneal washing cytology produced a positive result, laparoscopic gastrojejunostomy (modified Devine procedure) was performed to improve food passage, and S-1 (100 mg/body, days 1–14) plus paclitaxel (120 mg/body, days 1 and 15) was administered. Although the tumor was temporarily reduced in size, an abdominal computed tomography scan obtained after four courses of chemotherapy showed progressive disease. Thus, chemoradiotherapy (56 Gy, S-1: 60 mg/body, CDDP: 5 mg/body, days 1–5) was indicated. Marked tumor shrinkage and negative peritoneal washing cytological results were achieved. Curative gastrectomy with D2 lymphadenectomy was performed. We carried out distal gastrectomy and lymph node dissection, and the gastrojejunostomy produced as a gastric bypass in the previous operation was preserved. The patient has not suffered a tumor relapse in 4 years since the surgery.

**Conclusions:**

We surgeons increase a chance to perform R0 gastrectomy for stage IV gastric cancer following intensive chemotherapy and/or CRT. We should choose proper position of gastrojejunostomy in producing alimentary bypass for stage IV gastric cancer patients to facilitate curative surgery.

## Background

Intensive chemotherapy is indicated for stage IV gastric cancer, and its effects determine patient outcomes. Several reports have demonstrated the potential of S-1 as a treatment for advanced or recurrent gastric cancer in Japan [1]. Advanced gastric cancer in the lower third of the stomach often results in stricture of the gastric cavity and digestive symptoms such as appetite loss and vomiting. Gastrojejunostomy has been suggested to improve such symptoms [2]. The advent of new anticancer agents for gastric cancer has improved the response rate of the disease and has made it possible to destroy distant lesions [3–6]. Intensive chemotherapy might make it possible to perform R0 gastrectomy in part of patients with stage IV gastric cancer. In such cases, a gastrojejunostomy that was produced as a bypass might be used as a reconstruction route after curative gastrectomy.

We present a rare case of stage IV gastric cancer, in which curative surgery was performed after multidisciplinary treatment. A previous gastrojejunostomy was utilized as a reconstruction route during the curative procedure.

## Case presentation

**Fig. 1:**
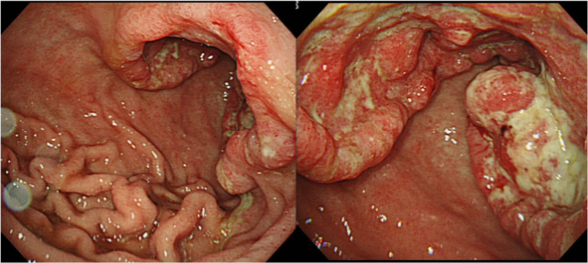
Endoscopic findings before the first operation. Gastroscopy revealed type 3 gastric cancer and pyloric stenosis in the lesser curvature of the antrum

An 80-year-old woman was admitted to Kagoshima University Hospital with a chief complaint of epigastralgia. Gastroscopy revealed type 3 gastric cancer in the lesser curvature of the antrum (Fig. 1). The pathological diagnosis of the biopsy specimen was poorly differentiated adenocarcinoma. Abdominal computed tomography (CT) revealed thickening of the antrum wall, 70 mm in diameter, and suggested that perigastric lymph node metastases were present. A blood test showed serious anemia (Hb 4.7 g/dl), and the patient’s carcinoembryonic antigen and carbohydrate antigen 19-9 levels were normal. The patient underwent a staging laparoscopy under general anesthesia, and perioperative washing cytology of her ascites produced a positive result. She was diagnosed with stage IV gastric cancer, and a gastrojejunostomy was produced in the middle third of her stomach to improve food passage. The status of gastric cancer was cT4a(SE)N2H0P0CY1 cStageIV (based on Japanese classification of gastric carcinoma [7]).

**Fig. 2:**
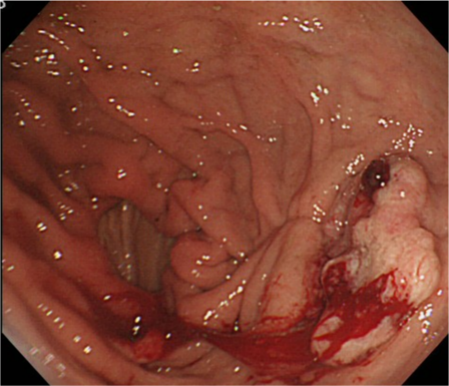
Endoscopic findings after chemotherapy. Regrowth of the gastric cancer was observed after four courses of chemotherapy

**Fig. 3:**
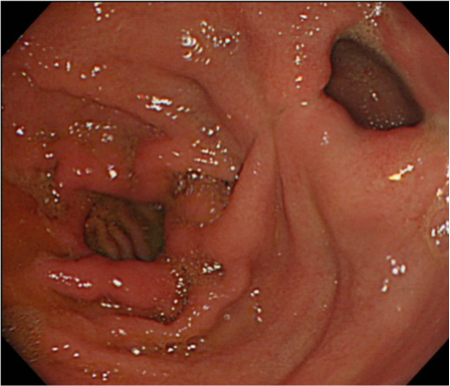
Endoscopic findings after chemoradiotherapy. Chemoradiotherapy resulted in marked tumor shrinkage

**Fig. 4:**
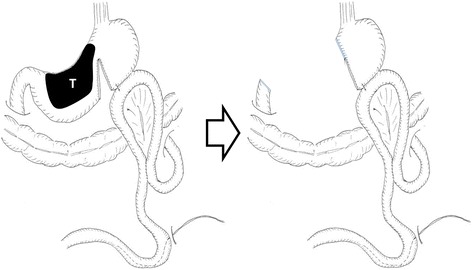
Distal gastrectomy. Gastrojejunostomy was reused to reconstruct the remnant stomach and jejunum

**Fig. 5:**
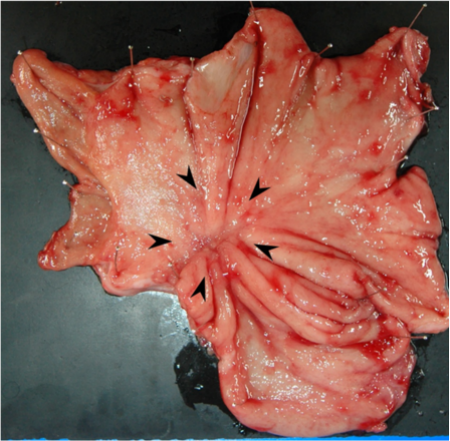
Resected specimen. The gastric lesion in the resected specimen was scarred (*arrow*)

Therefore, a combination of S-1 (100 mg/body, days 1–14) plus paclitaxel (120 mg/body, days 1 and 15) was administered. Although the tumor was temporarily reduced in size, gastroscopy and abdominal CT showed tumor regrowth after four courses of chemotherapy (Fig. 2). However, a preoperative peritoneal washing cytology produced a negative result (CY0) so chemoradiotherapy (CRT; total dose 56 Gy, S-1: 60 mg/body, CDDP: 5 mg/body, days 1–5) was started for the purpose of local control. The CRT resulted in marked tumor and nodal shrinkage, as determined by CT and gastroscopy (Fig. 3), and preoperative peritoneal washing cytology continued to produce negative results (CY0). Then, distal gastrectomy with D2 lymphadenectomy was performed with curative intent. The stomach was resected on the distal side of the gastric bypass, and the gastrojejunostomy was reused as a route for the reconstruction of the remnant stomach and jejunum (Fig. 4). Macroscopically, the gastric lesion in the resected specimen was scarred (Fig. 5). The pathological diagnosis was poorly differentiated adenocarcinoma, 10 × 5 mm, ypT3(SS), ly1,v-,N0,M0 ypStageIIA. Pathological examination revealed the proper muscular layer had disappeared, which had been replaced by the fibrous tissue in the center of scar. A few viable cancer cells were found in the tumor nest, so histological effect by the CRT was evaluated as grade 2 according to the Japanese classification of gastric carcinoma [7].

The patient’s postoperative course was uneventful. Because of a low level of white blood cell, she has been followed closely without adjuvant therapy. Four years after the operation, she is well, and the tumor has not relapsed.

### Discussion

The initial regimen used to treat this patient involved biweekly paclitaxel plus S-1. This regimen was originally established in our department [8, 9], and Nakajo et al. reported that it resulted in a response rate of 43.6% and a median survival time of 268 days, which are not inferior to those of the standard regimen for gastric cancer. Moreover, elderly patients are able to tolerate this approach and have been repeatedly treated with this regimen. In the present case, this chemotherapy regimen resulted in the disappearance of cytological positivity.

As the patient’s tumor was resistant to the first regimen, we chose to treat the patient with CRT after confirming that her distant metastases were under control. The use of second-line chemotherapy after the failure of first-line chemotherapy seems to be a common approach to the treatment of advanced gastric cancer. Hironaka et al. [10] suggested the clinical usefulness of second-line chemotherapy for stage IV gastric cancer. However, the response rate was lower than 21%, which was significantly lower than that for first-line chemotherapy.

CRT is included in the current standard regimen for advanced gastric cancer. Ajani et al. [11] reported that combined treatment involving radiotherapy and chemotherapy (5-FU plus cisplatin) in a neoadjuvant setting produced a R0 resection rate of 77% and a pathological complete response rate of 26%. Moreover, in Japan, Saikawa et al. [12] reported a markedly high response rate in a phase II study of CRT involving S-1 and cisplatin for unresectable gastric cancer. Finally, we found that CRT had a markedly beneficial histological effect on gastric cancer. It is suggested that CRT is a possible second-line therapy for advanced or recurrent gastric cancer. Therefore, multidisciplinary treatment involving CRT might be useful as a second-line treatment for locally advanced gastric cancer.

Three surgical strategies have been indicated for gastric cancer patients with cancerous obstruction: palliative gastrectomy, an alimentary bypass, and stent insertion. Patients that exhibit a good performance status are often indicated for gastrojejunostomy to produce an alimentary bypass [2]. Simple gastrojejunostomy often concomitant with delayed gastric emptying and bleeding from the tumor surface [13]. Devine et al. introduced the novel gastrojejunal bypass with a transection between the tumor and gastrojejunostomy to prevent such complications [14]. Schantz et al. [15] reported of modified Devine procedure with hemitransection of the stomach, which allows endoscopic examination. Therefore, modified Devine procedure seems to be suitable for stage IV gastric cancer patients planning to receive intensive chemotherapy and/or CRT like the current case. As a result, her pyloric stenosis improved, and she was able to receive treatment as an outpatient. The use of a laparoscopic approach during gastrojejunostomy is becoming increasingly common. In a previous series, we performed gastrojejunostomy after staging laparoscopy [16]. At the first surgery, we paid attention to keep a good margin from the tumor to produce gastrojejunostomy.

In the second surgical procedure, we simply resected the distal stomach and lymph nodes, and we were able to utilize the gastrojejunostomy produced during the first surgical procedure.

There have not been any previous reports about cases in which a previous gastrojejunostomy was utilized as a reconstruction route during distal gastrectomy in a patient with gastric cancer that had been treated with chemotherapy and/or CRT.

## Conclusions

We surgeons increase a chance to perform R0 gastrectomy for stage IV gastric cancer following intensive chemotherapy and/or CRT. We should choose proper position of gastrojejunostomy in producing alimentary bypass for stage IV gastric cancer patients to facilitate curative surgery.
